# Design, Synthesis, Characterization, and In Vitro Evaluation of a New Cross-Linked Hyaluronic Acid for Pharmaceutical and Cosmetic Applications

**DOI:** 10.3390/pharmaceutics13101672

**Published:** 2021-10-13

**Authors:** Sabrina Sciabica, Giovanni Tafuro, Alessandra Semenzato, Daniela Traini, Dina M. Silva, Larissa Gomes Dos Reis, Luisa Canilli, Massimo Terno, Elisa Durini, Silvia Vertuani, Anna Baldisserotto, Stefano Manfredini

**Affiliations:** 1 Department of Life Sciences and Biotechnology, University of Ferrara, Via L. Borsari 46, 44121 Ferrara, Italy; scbsrn@unife.it (S.S.); dre@unife.it (E.D.); smanfred@unife.it (S.M.); 2 Department of Pharmaceutical and Pharmacological Sciences, University of Padova, Via Marzolo 5, 35131 Padova, Italy; tafuro.giovanni.mds@gmail.com (G.T.); alessandra.semenzato@unipd.it (A.S.); 3 Macquarie Medical School, Department of Medical Sciences, Faculty of Medicine, Health and Human Sciences, Macquarie University & Woolcock Institute of Medical Research, Sydney 2037, Australia; daniela.traini@mq.edu.it (D.T.); dina.silva@sydney.edu.au (D.M.S.); larissagomesreis@yahoo.com.br (L.G.D.R.); 4 Istituto Ganassini S.p.A., Via Carlo Boncompagni, 63, 20139 Milano, Italy; l.canilli@ganassini.it (L.C.); dir.tecnica@ganassini.it (M.T.)

**Keywords:** hyaluronic acid, cross-linking, biocompatibility

## Abstract

Hyaluronic acid (HA), an excellent biomaterial with unique bio properties, is currently one of the most interesting polymers for many biomedical and cosmetic applications. However, several of its potential benefits are limited as it is rapidly degraded by hyaluronidase enzymes. To improve the half-life and consequently increase performance, native HA has been modified through cross-linking reactions with a natural and biocompatible amino acid, Ornithine, to overcome the potential toxicity commonly associated with traditional linkers. 2-chloro-dimethoxy-1,3,5-triazine/4-methylmorpholine (CDMT/NMM) was used as an activating agent. The new product (HA–Orn) was extensively characterized to confirm the chemical modification, and rheological analysis showed a gel-like profile. In vitro degradation experiments showed an improved resistance profile against enzymatic digestions. Furthermore, in vitro cytotoxicity studies were performed on lung cell lines (Calu-3 and H441), which showed no cytotoxicity.

## 1. Introduction

Over the past decade, hyaluronic acid (HA) has proven to be one of the best materials for application in various fields including pharmaceutical, food, and cosmetic applications, mostly due to its safety profile and unique physicochemical, biological, and viscoelastic properties.

HA is commonly used in ophthalmology to protect and lubricate the delicate eye tissues, supplement vitreous humour, and particularly in cataract surgery to replace lost vitreous fluid and to maintain the space and avoid the collapse of the eye tissue during the operation. On the other hand, HA is the main ingredient of many artificial tears that are produced with HA at different molecular weights and different polymer concentrations. Therefore, HA-based ophthalmic products are completely biocompatible and do not trigger foreign body reactions [1]; eye drops with hyaluronan derivatives with improved mechanical and biological properties have recently been formulated [2].

Because HA has both structural and regulatory functions in the processes of wound repair and re-epithelialization [3] as well as in the stimulation of collagen production, it has been considered as an appropriate candidate to support skin tissue regeneration. Therefore, it is often included, alone or in combination with other therapeutic agents, in topical formulations for the treatment of skin irritations and wounds including abrasions and burns as well as post-surgical, metabolic, and vascular ulcers. Generally, these products are used not only in the dermatological field but also in ophthalmology, otolaryngology rhinology, and dentistry [4]. Cross-linked HA hydrogel films have also been shown to accelerate the wound-healing process by promoting the re-epithelialization that typically occurs after damage [5]. Recently, biomimetic injectable biogels have been designed and developed via the cohesive molecular assembly of a network of polysaccharide polymers to provide HA with in situ gelling properties and avoid implant surgery [6].

Considering that hyaluronic acid is a natural anti-inflammatory and antioxidant, it represents a multifunctional agent for the treatment of certain lung diseases including airway diseases with a predominant inflammatory component such as rhinosinusitis, asthma, chronic obstructive pulmonary disease, cystic fibrosis, and primary ciliary dyskinesia [7,8,9,10]. All of these lung diseases are characterized by periodic or chronic inflammatory processes as a result of releasing inflammatory cytokines from the cells such as IL-6 and IL-8. These inflammatory mediators result in an increase in reactive oxygen species (ROS) and hence an increase in oxidative stress in the lungs [11] that play a key role in the regulation of wound healing. Recently, the use of polymers such as HA has also been reported in airway treatment because of their unique ability to retain water and regulate fluid balance in the lung interstitium. Due to its anti-inflammatory properties and tissue repair properties, the integration of exogenous high-molecular-weight hyaluronic acid can provide important results in reducing inflammation and, consequently, disease symptoms [12]. The use of HA, as a therapeutic agent for the treatment of airway pathologies, is interesting because it opens the possible development of a new class of therapeutic agents resulting from the combination with molecules active for application in pulmonary therapy.

It is known that the concentration of HA in the skin decreases drastically with age, causing the formation of wrinkles [13,14]. For this reason, HA-based dermal fillers (DF) in recent years have aroused wide interest in the field of cosmetic surgery to restore lost volume and correct facial imperfections of the dermis [15]. There are several types of HA-based DFs on the market; they are usually cross-linked HA-based and show a structured network behaviour designed to persist in the body. The corrective effect is reversible and lasts from three to 24 months [16]. HA is also found in sunscreen products for its potential ability to protect the skin from harmful radiation due to its possible scavenging properties [17,18]. Due to its high hygroscopicity, HA can retain large quantities of water, providing hydration, conferring its application as a physical barrier depending on the molecular weight.

Although HA is an excellent biomaterial, many of its potential applications are limited due to its short half-life since after interaction, due to the CD44 receptor present on a variety of cell membranes, it is internalized and rapidly degraded by hyaluronidases [19,20].

Chemical modifications of HA can be designed to enhance the half-life and modulate the desired therapeutic action while maintaining linear HA original properties, such as biocompatibility, biodegradability, and mucoadhesiveness [21]. To achieve this, derivatization strategies involving mainly two sites of HA functional groups have been used: the carboxylic group on the D-glucuronic acid and, more frequently, the hydroxyl group present on the *N*-acetyl D-glucosamine.

The first example of a reaction is conjugation, in which a molecule is grafted onto a functionality of the polymer; the second type is the cross-linking of different chains through covalent bonds, using a chemical cross-linker [1].

Usually, the conjugation reaction is used to obtain the therapeutic effect [22] and can be used to add new reactive groups on the HA scaffold or to obtain a drug vehicle [1,23,24]. Cross-linking instead is mostly used to improve mechanical and rheological properties with consequent reduction of the degradation rate [25]. 

Known methods generally use synthetic cross-linkers including divinyl sulfone (DVS), 1,4-butanediol diglycidyl ether (BDDE) [26], and carbodiimide [1]. These cross-linkers can form a three-dimensional reticular structure with greater mechanical strength but have poor biocompatibility characteristics that can cause adverse reactions [27,28,29]. 

Therefore, several studies are focused on obtaining new and safe hydrogels to increase biocompatibility and biodegradability [30,31]. Specifically, recent studies have reported low (LMW) and high-molecular-weight HA (HMW) cross-linked by urethane bond formation, using a diisocyanate (BIED), which has a better profile than commonly used aryl di-isocyanates. These novel cross-linked hydrogels have shown potential use for vaccine administration (LMW HA) and cell encapsulation to reduce immune rejection of transplanted cells (HMW HA) [32]. Furthermore, this new material showed interesting bacteriostatic properties towards *Staphylococcus (S.) aureus* [33]. Another field of interest concerns the potential applications of innovative HA-based drug delivery systems in inflammatory skin diseases. HA-based nanoparticles and pluronic hydrogels were observed to enhance drug permeation through intact skin for the treatment of atopic dermatitis [34]. Furthermore, the use of gelatine and hyaluronic acid as the main materials for the preparation of absorbable hydrogel regeneration membranes has been proposed [35].

In this work, a new cross-linked HA has been developed, through a cross-linking technique that involves the formation of a reactive intermediate and the use of a new cross-linker, an ornithine methyl ester, a derivative from the natural and biocompatible aminoacidic precursor ornithine. Ornithine is a molecule naturally present in the human body where it is part of the Natural Moisturizing Factor (NMF) [36] and is therefore considered biocompatible and non-toxic. Ornithine is used in the nutraceutical and cosmetic field as a functional ingredient as it is recognized as an anti-ageing, immunostimulant, and promoter of tissue repair. Its chemical structure attracted our attention because of its use as a crosslinker and a possible carrier of active moieties through its homo-bifunctional amino residues. Due to the safety and beneficial effects of ornithine and HA, these two components were chosen for the synthesis of a new and innovative HA-based biopolymer with enhanced bioactivity, mechanical properties, and reduced toxicity. The availability of these innovative raw materials prompted us to investigate potential cosmetic, pharmaceutical, and nutraceutical applications, not only of the molecule as is, as active ingredients, but also as possible carriers of active molecules to the skin, as previously described [37,38].

## 2. Materials and Methods

### 2.1. Materials

Sodium salt Hyaluronate (HA) isolated from *Streptococcus equi* with average Mw 1.2 MDa was purchased from Caldic (Origgio, Varese, Italy). Ornithine methyl ester (H-Orn-OMe^.^ 2HCl) was purchased from Bachem. 4-methylmorpholine (NMM), NaCl, 0.025 M sodium tetraborate, sulfuric acid, and acetonitrile were purchased from Sigma–Aldrich. 2-chloro-dimethoxy-1,3,5-triazine (CDMT) purchased from TCI. The phosphate buffer saline (PBS) was purchased from Sigma–Aldrich (Sigma-Aldrich SRL, Milano, Italy).

### 2.2. Cross-Linked HA–Orn

#### 2.2.1. Processing Parameters

The synthesis of the product was conducted initially with EDC/NHS and then with CDMT/NMM as condensing agents to set up the ideal synthetic conditions to obtain an HA with a high degree of cross-linking and good yield. For the EDC/NHS route, several increasing stoichiometric ratios were used between HA: EDC: Ornithine but with poor results. From the scale-up study conducted, CDMT, in association with NMM, was found to be the most potent activator. Therefore, we switched to the latter as a condensing agent, leading to the synthesis of Ornithine cross-linked polymer, initially using an excess of both condensing agent and amino acid with a stoichiometric ratio of 1:4.5:3 between HA:CDMT/NMM:Ornithine. This ratio was subsequently reduced to 1:3:1.5 as it was able to offer the same product in terms of crosslinking and yield, avoiding reagent waste. The degree of cross-linking was directly proportional to the number of molecules forming double links, which play an important role in the viscoelastic properties of HA. It is important to evaluate the desired degree of cross-linking for the development and commercialization of new HA products, especially for biomedical and cosmetic applications. A polymer with a high degree of cross-linking corresponds to a slower degradation rate, degrading slower in the body. The result of chemical cross-linking is a three dimensional HA hydrogel that could retain water within its cross-linked network but does not dissolve readily in water. This allows for greater resistance resulting from a reduction in the access of digestive enzymes in the polymeric network and the consequent hydrolytic cut.

#### 2.2.2. Synthesis of Cross-Linked HA–Orn

HA sodium salt (264 mg; 0.66 mmol of carboxylate group) was uniformly dissolved in 52 mL of deionized water before the addition of 35 mL of acetonitrile. The homogeneous solution was cooled in an ice bath for 0.5 h, and 347.5 mg of CDMT (1.98 mmol) was added. After 1 h, 261.9 mg of Orn-OMe and 2 HCl (0.99 mmol) were added. The pH value was adjusted to 4 with HCl 0.5 M, and then 217.8 μL of NMM (1.98 mmol) was added and the reaction was stirred (~400 rpm) overnight. The HA–Orn product was then purified using a dialysis membrane tube with an Mw cut-off of 3.5 KDa (Thermo Fisher Scientific, Italy) against H_2_O for 24 h, followed by NaCl (0.1 M) for two days and finally against H_2_O for two days. The purified HA–Orn was lyophilized using a Lio-5P lyophilizer (Vetrotecnica S.r.l., Padua, Italy), resulting in a white powder with a 73% (280 mg) yield. NMR spectra were registered with a spectrometer Mercury Plus-400 (Varian) at 400 MHz at room temperature. HA–Orn ^1^H NMR (400 MHz, D_2_O) δ ppm: 4.43 (m, 3H, 2 O–CH–O anomeric carbon of HA; –CH– α of Ornithine), 3.97–3.09 (m, 15H, C–CH–O of HA; –OCH_3_ of Ornithine), 2.92 (m, 2H, CH_2_ δ of ornithine), 1.97 (s, 3H, CO–CH_3_–N of HA), 1.80–1.56 (m, 4H, –CH_2_ β and –CH_2_γ of ornithine).

### 2.3. Chemical–Physical Characterization of HA–Orn

#### 2.3.1. Fourier-Transform Infrared Spectroscopy (FTIR)

FTIR measurements were performed on dry powder samples using an FTIR spectrophotometer (PerkinElmer Spectrum 100) and an ATR method (attenuated total reflectance) scanned from 4000 to 650 cm^−1^, at room temperature. The samples were placed directly on the crystal, and FTIR spectra were obtained after eight scans with a resolution of 1 cm^−1^. IR (cm^−1^): 3267, 1730, 1617,1640, 1515, 1409, 1010.

#### 2.3.2. Differential Scanning Calorimetry (DSC)

The heat transition of the samples was determined using a differential scanning calorimeter DSC60A (Shimadzu Corporation Kyoto, Kyoto, Japan) calibrated with a pure indium standard. Samples (2 to 6 mg) were placed in hermetically sealed aluminium pans and heated from 25 °C to 300 °C at 10 °C/min. Measurements were performed in an inert nitrogen atmosphere purged at a flow rate of 45.0 mL/min. The endothermic and exothermic peaks were elaborated using TA-60 WS system software (Shimadzu corporation Kyoto, Japan).

#### 2.3.3. Scanning Electron Microscopy (SEM)

Lyophilized samples of HA and HA–Orn were observed using a field emission scanning electron microscope (Zeiss EVO 40XVP, Carl Zeiss Pty Ltd., Oberkochen, Germany), with a voltage acceleration of 20 kV. Samples were deposited on carbon sticky tabs, analyzed in variable pressure mode, scanned, and photographed randomly.

#### 2.3.4. Swelling Measurement

The swelling degree (SD) of HA–Orn is defined as the ratio between the weight of the swollen gels (Ws) after prolonged dialysis in aqueous media at different pH values vs. the weight of the dry gels (Wd). Dry lyophilized samples (100 mg) were exactly weighed before loading in a dialysis tube with 300 mL of PBS at pH values equal to 4.5, 6.5, and 9, and deionized H_2_O at 25 °C. The weight of each swollen sample after 24 h was determined to calculate SD according to Equation (1):

% SD = Ws/Wd(1)

#### 2.3.5. Rheological measurement

The rheological analyses were performed using a Physica MCR-101 rheometer (Anton Paar GmBH, Graz, Austria), equipped with PP50-P2 parallel plate geometry with serrated surfaces. The gap between the surfaces of the plates was fixed at 1 mm. A Peltier system ensured a temperature of 23 ± 0.05 °C. Before the measurements, all samples were maintained at the initial temperature for 1 min. A Controlled Shear Rate (CSR) test was conducted in triplicate under continuous flow conditions, recording the viscosity (η) values of the samples at increasing shear rates ranging from 0.001 to 1000 s^−1^. The flow curves obtained were fitted with the Carreau–Yasuda mathematical model (Equation (2)), which describes the shear-thinning behavior and allows the calculation of the zero-shear viscosity (η0) i.e., the material’s viscosity at rest [39]:


(2) $$\;\tau =\eta \infty \overset{•}{\gamma }+\frac{\left(\eta 0-\eta \infty \right)\overset{•}{\gamma }}{{\left[1+{\left(\lambda \overset{•}{\gamma }\right)}^{2}\right]}^{\frac{1-n}{2}}}$$

The viscoelastic behavior under oscillatory flow conditions was measured using the trends of the storage (G′) and the loss (G″) modules [40]. An Amplitude Sweep (AS) test was performed at a fixed value of frequency (1 Hz), varying the strain (γ) from 0.01 to 1000% to identify the Linear Viscoelastic Region (LVER). A Frequency Sweep (FS) test was conducted at a fixed value of strain from LVER, increasing the values of frequency in a range from 10 to 0.01 Hz to study the materials’ inner structure and physical stability [41]. The G* parameter is an indicator that measures some physical–mechanical properties such as the firmness and the flexibility of the material structure (Equation (3)).


(3) $$\left|G*\right|=\sqrt{{G^{\prime }}^{2}+{G^{\prime \prime }}^{2}}$$

The damping factor (tan δ), calculated from the ratio between the loss modulus G″ and the storage modulus G′, was used to describe the predominant component between solid–elastic or liquid–viscous.

#### 2.3.6. Water Content

The relative moisture sorption of freeze-dried HA and HA–Orn after exposure to different humidity levels (0–90% RH) was analyzed by Dynamic Vapour Sorption (DVS, DVS-1, Surface Measurement Systems Ltd., London, UK). Each sample was placed in an aluminum pan and exposed to two cycles of 0–90% relative humidity (RH) at 25 °C, at 10% RH increments. The equilibrium moisture content in each humidity phase was determined as a change in the ratio of mass versus time (dm/dt) of 0.0005% min^−1^.

### 2.4. Enzymatic Degradation Test

#### 2.4.1. Sample Disc Preparation

HA and HA–Orn were prepared for the enzymatic degradation test by forming solid discs. Briefly, 1 mL of an aqueous solution (1.5% *w*/*v*) of each sample was poured into spherical molds with a diameter of 10 mm and then lyophilized. The dehydrated samples were then pressed to form compact discs, weighing 15 mg and 1 mm thick.

#### 2.4.2. In Vitro Degradation

A stock solution of hyaluronidase from bovine testes (type IV-S powder, 1045 units/mg, lot SLCC9109, from Sigma Aldrich) was prepared at a concentration of 50 units/mL in PBS. The discs of each sample in this enzymatic solution were kept under agitation at 37 °C for different test times (up to 24 h) to verify the glucuronic acid released. To verify the absence of degradation phenomena due to temperature, a control was carried out on each test sample without HAse at the same temperature (data not shown).

#### 2.4.3. Carbazole Assay

At pre-determined time points, 200 μL of supernatant was withdrawn and added to 3 mL of 0.025 M sodium tetraborate in tubes containing sulfuric acid. The samples were vortexed for 10 s and then boiled for 10 min at 100 °C. After cooling, 100 μL of 0.0125% carbazole reagent in EtOH (absolute) was added to the samples, and the reaction was started by boiling for further 15 min. The amount of GlcA produced after degradation was monitored by absorbance reading with a spectrophotometer UV-31 SCAN ONDA (Giorgio Bormac S.r.l., Carpi (Modena), Italy) at 523 nm. A blank control was prepared with phosphate buffer only. 

### 2.5. Biological Assays

#### 2.5.1. Cells Culture

Calu-3 and NCI-H441 cell lines (carcinoma-derived epithelia, ACTT, Rockville, MD, USA) were chosen as a model of respiratory cells due to their ability to grow in an air–liquid interface and produce tight junctions and mucus, similar to the physiological conditions observed in the lung. Both cell lines were cultured in 75 cm^2^ flasks and were maintained in humidified 95% air, 5% CO_2_ atmosphere, at 37 °C. Calu-3 was cultured in Dulbecco’s Modified Eagle’s Medium/F-12 supplemented with 1% (*v*/*v*) non-essential amino acids, 1% (*v*/*v*) 200 mM L-glutamine solution, and 10% (*v*/*v*) fetal bovine serum (FBS), while H441 cells were cultured in RPMI-1640 medium, supplemented with 10% (*v*/*v*) FBS. Media were changed 2–3 times per week until confluency; then cells were passaged using trypsin at 1:3 and 1:7 ratios for Calu-3 and H441 cells, respectively. To establish an air–liquid interface (ALI), both cell lines were seeded at 3 × 10^4^ cells per well of a Transwell polyester insert containing 100 µL in the apical chamber and 600 µL in the basal chamber. The medium from the apical chamber was removed 24 h after seeding and every day afterwards until an ALI was achieved, while the medium from the basal chamber was replaced every second day up to 14 days of culture. For H441, a differentiation medium was used on the basal chamber from day 2 from seeding the cells consisting of RPMI-1640 medium supplemented with 200 nM dexamethasone (Sigma) and 1% (*v*/*v*) insulin-transferrin-selenium supplement (100×, Gibco, Sidney, Australia).

#### 2.5.2. MTS Assay for Cytotoxicity

Cells were seeded at a density of 50 × 10^4^ cells/well in a 96-well plate. After 24 h, the media were removed and replaced with 100 µL of the raw materials prepared in HBSS. After 24 h incubation, 20 µL of MTS reagent was added to the wells and read after 3 h at 490 nm. The solutions were prepared as a serial dilution in the range of 0.009–0.30% (*w*/*v*) for HA and HA–Orn. DMSO 20% was used as the control for cell death.

### 2.6. Statistical Analysis

All the results are reported as the mean ± standard error of at least three separate determinants. The statistical software GraphPad Prism (version 8.2.1) was used to test for significance by performing an unpaired t-test for significance in each experiment. Significance was determined as *p* < 0.05.

## 3. Results

### 3.1. HA–Orn Cross-Linked Synthesis

The HA cross-linked product was successfully prepared by amidation of the carboxyl groups through a synthetic procedure adapted from the Bergman method [42] using the amino acid Ornithine methyl ester as a cross-linker to form diamide bonds between the chains of HA. CDMT, in association with NMM, was found to be the most efficient activator. The synthesis of HA–Orn was carried out at room temperature with an excess of both activating agent and the amino acid at a ratio of HA: CDMT: amino acid of 1: 3:1.5.

**Figure 1:**
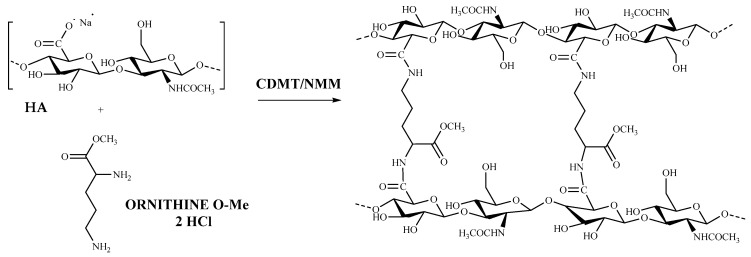
Synthetic scheme of HA crosslinked with the Ornithine methyl ester.

The reaction initially involved the activation of the carboxylic acid with CDMT to form the intermediate, activated HA; then, NMM was added to the mixture in equimolar ratio to CDMT to neutralize the chloride ions formed (Figure 1). A purification step by exhaustive dialysis was conducted until the resulting hydrogel was completely purified from any detectable residual cross-linker agent to finally obtain a white dry powdery product.

### 3.2. Physico–Chemical Characterization Methods

#### 3.2.1. ^1^H-NMR

**Figure 2:**
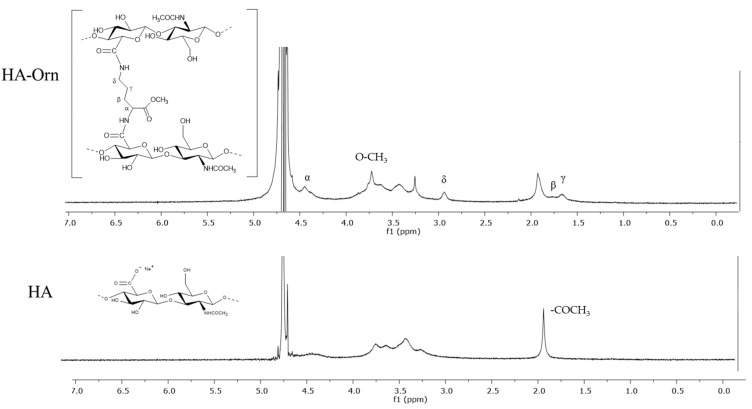
^1^H-NMR spectrum of native HA and cross-linked HA–Orn recorded in D_2_O.

^1^H-NMR spectroscopy (Figure 2) was used for the characterization of the chemical modification that occurred on native HA. Specifically, the degree of modification was quantified by calculating the integration of a signal belonging to the cross-linker to the characteristic peak of the native HA at 1.9 ppm that was specific for the protons of the methyl proton group of *N*-acetyl glucosamine.

The spectra of hyaluronic samples gave enlarged signals due to the viscosity of the product in the solution. The peaks of the methyl groups of the Ornithine side chain were selected since they did not overlap with the peaks of the native HA, allowing for an easy calculation of the degree of modification. 

The HA–Orn spectrum displayed three characteristic peaks: two wide signals at 1.56 ppm (-CH_2_ γ) and 1.72 ppm (-CH_2_ β) and the methoxy group at 2.89 ppm.

In all spectra, the multiplet of signals in the region from 3.2 to 3.9 ppm corresponded to the protons of the HA component. However, HA also showed a large signal at 4.4 ppm related to the two anomeric protons. In the spectra of HA–Orn, this signal overlapped with the signals of the methyl groups in α at 4.5 ppm. From the calculation of the ratio between the two reference signals, it was found that the degree of modification was estimated at around 35% for HA–Orn; these values corresponded to the amount of Ornithine cross-linked with native HA.

#### 3.2.2. IR Spectroscopy

**Figure 3:**
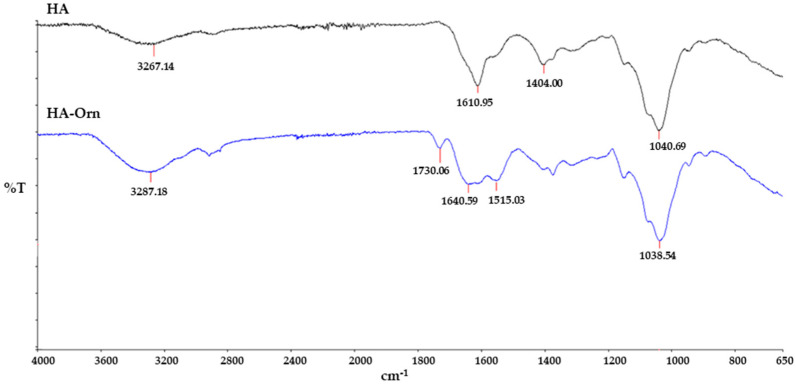
FTIR spectra of native HA and cross-linked HA–Orn.

Figure 3 shows the difference in the IR spectra between HA and HA–Orn. Native HA showed main absorption bands at 3267 cm^− 1^ corresponding to O–H and N-H group stretching; a double band at 1617 cm^−1^ and 1409 cm^−1^ was attributed to the asymmetric and symmetric C=O stretching modes of the planar carboxylate groups, and a band at 1010 cm^−1^ indicated C-OH stretching vibrations. HA–Orn profiles showed (1) the appearance of a C=O amide band at 1640 cm^−1^, which corresponded to the new amide bonds from the cross-linking reaction; (2) the band related to the carboxylate groups at 1600–1400 cm^−1^ disappeared with an increase in the intensity of the NH group peak at about 3200 cm^−1^; furthermore, a NH bending band at 1515 cm^−1^ was observed; and (3) another new band was evident at around 1730 cm^−1^ relative to the carbonyl group of the methyl ester on the amino acid. These results agree with the expected product after the cross-linking reaction, providing further evidence of the desired bond formation.

#### 3.2.3. Thermal Analysis: DSC

Chemical cross-linking leads to chemical–physical changes that can be correlated with different in vitro and in vivo behaviours with respect to native HA. To study how the chemical modification could influence the interactions between the polymer chains and the water content, the thermal behaviour of HA–Orn was investigated by differential scanning calorimetry [43], a thermal analysis able to provide information on their hydration and thermal resistance properties [44,45,46]. The DSC technique is based on the different temperature demands between the reference and the sample as well as the measurement of the heat absorbed or released by the system when the temperature changes following structural changes.

**Figure 4:**
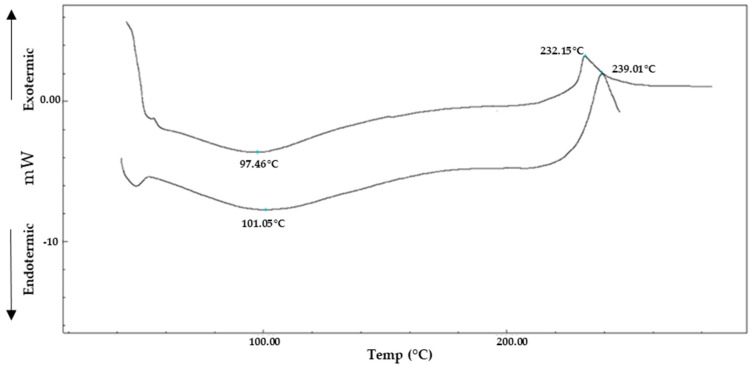
DSC thermograms of native HA and HA–Orn.

The HA profile (Figure 4) showed two peaks: an endothermic peak around 101 °C, suggested the temperature of dehydration, followed by an exothermic peak attributable to the sample decomposition temperature at 238 °C. A similar profile was shown by HA–Orn but with lower onset temperature values: a large endothermic peak was observed at 97.4 °C associated with the loss of moisture (residual after lyophilization) followed by an exothermic peak at a lower temperature (232 °C), corresponding to the decomposition of HA–Orn.

The shift of the endothermic peak in the cross-linked product reflects lower water retention. Furthermore, the exothermic peak of HA–Orn was present at a lower temperature of (~7 °C) than native HA due to the presence of amino acid in the new structure. These observations showed that the cross-linking produced a new material with a different structural organization from the originator.

#### 3.2.4. Scanning Electron Microscopy (SEM)

**Figure 5:**
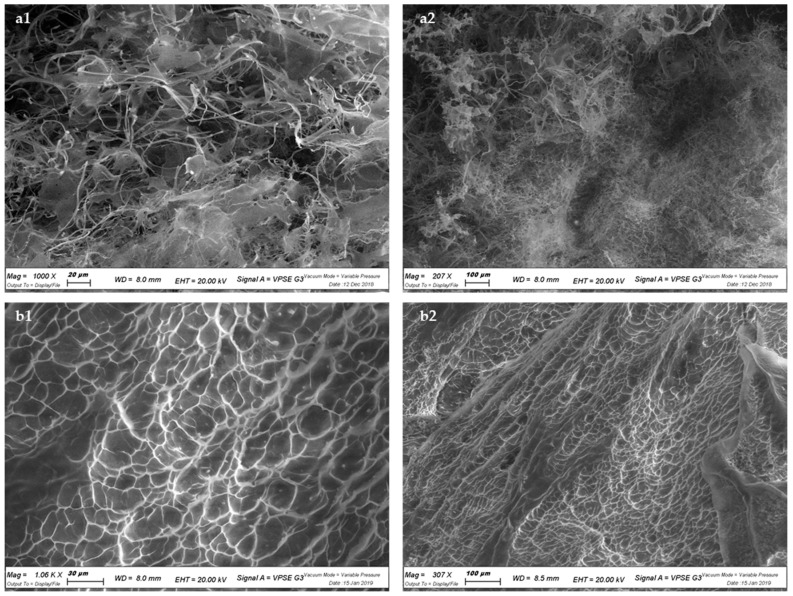
SEM images of HA (panels **a1**,**a2**) and HA–Orn (panels **b1**,**b2**). SEM Scale bar: 20 µm (panel **a1**), 100 µm (panels **a2**,**b2**), 30 µm (panel **b1**).

In Figure 5, SEM surface images of the native HA and HA–Orn cross-linked are shown. Native HA images (Figure 5, panels a1 and a2) showed a filamentous and irregular structure, while HA–Orn novel hydrogel showed a well-defined scaffold-like structure. HA–Orn (Figure 5, panels b1 and b2) showed a firmer and evenly distributed structure with small pores. 

Compared to native HA, HA–Orn is characterized by a network structure. From the SEM images, interconnectivity between the solid structures seems to emerge as a characteristic of the HA–Orn product. It can be hypothesized that these structural characteristics could allow the new polymer, when placed in an aqueous medium, to behave like a hydrogel [47].

#### 3.2.5. Swelling Ratio Measurement

**Figure 6:**
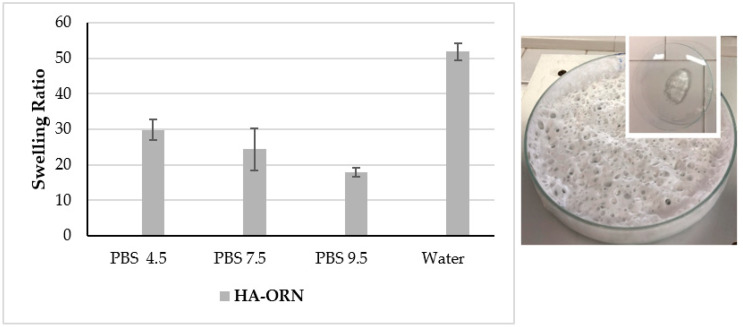
Left: Swelling ratio of HA–Orn at different pH values (*n* = 3; mean ± StDev) in PBS. Right: the behavior of HA–Orn before and after the addition of water.

Control of the rate and extent of hydration of the material is necessary to promote cell adhesion and subsequent prolonged activity; crosslinking strategies are used to achieve this goal [48], with high swelling values linked to a lower cross-linking density [1]. In particular, when cross-linked, HA can form a three-dimensional network capable of swelling and modulating the release of the loaded active ingredients, such as antibacterial molecules [49], anti-inflammatory substances [50], proteins, and antibodies [51]. Figure 6 presents the 24-h swelling of the cross-linked HA–Orn samples at 25 °C for a range of pH values, which are representative of some biological fluids (4.5–9.5) [52,53]. Linear HA uncross-linked dissolves rapidly when immersed in an aqueous medium, and therefore the swelling could not be measured.

The results obtained are in agreement with the swelling values typical of hydrogels. The swelling property of HA–Orn decreases with increasing pH, suggesting the influence of the ionic strength of the buffer. The highest swelling of HA–Orn was observed when the sample was immersed in distilled water, possibly due to electrostatic repulsion between the anionic charged groups of the carboxyl group (pKa 2.9), which allows a greater distention of the derivatized HA chains. The difference in pH can easily explain the different swelling behaviours, with the reduced swelling at basic pH possibly due to the lack of ionization of the carboxyl groups. The results suggest that the hydrogel exhibits a certain degree of pH reactivity, a phenomenon that could be exploited to modulate the swelling degree of cross-linked hydrogels, potentially useful for drug release applications.

#### 3.2.6. Rheology

**Figure 7:**
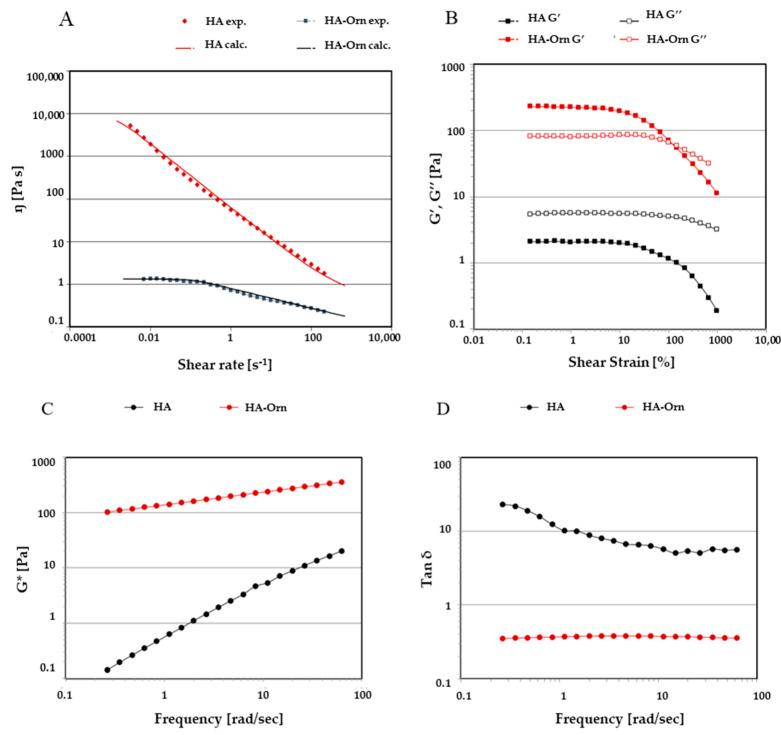
The trend of viscosity values (panel **A**), G′ and G″ moduli (panel **B**), complex moduli (G*) (panel **C**), and damping factor (tan δ) (panel **D**).

A Controlled Shear Rate test was conducted on the cross-linked hyaluronic acid dispersion prepared at a concentration of 2% *w*/*w* of raw material to analyze the viscosity as a function of the shear rate, ranging from 0.001 to 1000 s^−1^. HA–Orn showed a shear-thinning behavior since the viscosity values decreased as the shear increased (Figure 7, panel A). HA–Orn reached higher viscosity values than HA, due to strong interactions between the material’s cross-linked chains and the solvent, forming a three-dimensional network, in which the aqueous solvent is trapped. Indeed, the introduction of chemical crosslinking between the polymer chains leads to a permanent stable network where the individual chain’s flow unit is lost, and consequently, the intrinsic mobility of the resulting molecule undergoes a substantial reduction. The calculated viscosity at rest (η0) for HA–Orn was 9578.38 Pa·s. The viscosity rapidly decreased as a function of the shear rate, as shown in Figure 7, which means that the polymeric chains started to align toward the direction of the flow. HA dispersion showed a viscosity value more than three orders of magnitude lower than that of HA–Orn. The calculated value of η_0_ for HA was 1.33 Pa·s.

The viscoelastic behaviour of both samples was investigated under oscillatory flow conditions. By increasing the strain values from 0.01 to 1000%, at a fixed oscillation frequency of 1 Hz, an Amplitude Sweep test was conducted. This test allowed identification of the Linear Viscoelastic Region (LVER) in which the elastic G′ and the viscous G″ moduli were not dependent on strain showing a constant trend (Figure 7, panel B).

According to the flow condition experiment, the two samples showed very different viscoelastic properties. The HA solution had a liquid-like behaviour with G” always dominating over G′ for the entire range of amplitude strain investigated. On the contrary, HA–Orn dispersion was characterized by high moduli values, with G′ over G″ in the LVER, indicating a predominantly elastic behaviour of the material due to the highly swollen network formed in water. After this region, increasing the amplitude strain, G′ progressively decreased until it intercepted, at just about 100% of the strain, the viscous modulus G″ in the critical strain point (γG′=G″), with consequent inversion of the moduli, thus indicating the breakage of the microgel structure.

Figure 7 (panel C) shows G* trends as a function of the oscillation frequency applied to the sample. As expected, HA–Orn showed higher values of G* (measured between 100 and 100 Pa) than HA, almost independent from the applied frequency. Instead, the G* values for HA were strongly correlated with the changes in frequency, with the curve decreasing with decreasing values of frequency. The tanδ values > 1 indicated a liquid-like behaviour of HA dispersion and a greater dissipative capacity, while the HA–Orn sample (Figure 7, panel D) had values < 1, indicating a prevalence of the elastic modulus. This rheological profile describes a typical weak-gel pattern, where the storage G′ modulus dominated over the loss G″ modulus throughout all the range of frequencies investigated. Overall, the elastic components always dominated over the viscous component, probably due to the presence of a high crosslinking degree in the hydrogel system.

#### 3.2.7. Dynamic Vapour Sorption

To characterize the stability of the new polymeric network, a study was conducted on HA and HA–Orn under different humidity conditions (0–90% relative humidity, RH) using dynamic vapor absorption (DVS). The resulting moisture absorption–desorption isotherms (Figure S1) confirmed the typical trend of a hygroscopic HA-based polymer. In agreement with the literature [54,55], HA showed the water content increased linearly up to 29.3% in response to an RH increase from 0 to 70%. From 70–90% RH, the moisture absorption increased rapidly, leading to a water content of 63.1%. This rapid increase in moisture absorption is likely due to the hydrogel’s remarkable ability to retain moisture under high RH conditions. HA–Orn cross-linking showed a final water content value of 37.1% at 90% RH. This behavior is in accordance with the characteristics of the novel cross-linking process that leads to a reduction in the polymer’s ability to absorb water [56,57].

Desorption curves of HA and HA–Orn (Figure S1) showed a similar profile to the absorption curves. Minimal hysteresis phenomena were observed, indicating that despite being sensitive to moisture, this effect was reversible. These results indicate that, despite the modification due to the crosslinking process, the HA–Orn polymer preserved part of the water-retention capacity of its precursor HA and therefore its properties. At the same time, the cross-linking process made it less susceptible to water absorption, which excludes permanent alterations in the polymer structure.

### 3.3. In Vitro Degradation

To provide a cross-linking efficiency profile and information about the biodegradability of the new hydrogel, it is essential to define the field of application. In general, in vitro degradation offers preliminary proof of the resistance that the new biomaterial has acquired with the crosslinking process. As the concentration of hyaluronidases is tissue-specific, it is difficult to predict the real concentration in vivo. In this study, the concentration of hyaluronidases used was 50 units/mL, in line with most of the concentrations used in the literature [58,59] for the same assay (10–100 units/mL); the molecular weight of the samples under examination was also considered. The chosen concentration proved to be an excellent compromise to be able to observe the degradation profile of both HA and its cross-linked derivative over a sufficiently long period of 24 h. The in vitro degradation assay was performed by Bovine testes hyaluronidase as a hydrolytic enzyme to mimic the in vivo conditions where the enzyme cleaves the β 1–4 linkages, yielding fragments with *N*-acetylglucosamine (NAG) at the reducing terminus and glucuronic acid at the nonreducing end. The amount of glucuronic acid (GlcA) released from samples was tested using the Carbazole colorimetric assay according to the method originally described by Bitter et al. [60] based on the quantitative colorimetric reaction through two consecutive steps: hydrolysis of the HA supernatant in sulfuric acid to generate GlcA and NAG monomers and development of the colorimetric reaction between GlcA and Carbazole.

The amount of glucuronic acid (GlcA) released from the native HA sample was shown to be greater than the cross-linked amount (Figure S2). Its maximum value was reached at 240 min (159.532 μg/mL) and then remained almost unchanged, which means that the GlcA produced by the degradation of 15 mg of HA reached its maximum value. Complete degradation did not occur up to 24 h incubation for the cross-linked hydrogel. Cross-linked HA–Orn instead showed insoluble fractions after 24 h, with a released GlcA value of 113.5 μg/mL. HA–Orn after 2 h of degradation released 15.9% GlcA, while the native HA released 57.2% (91.42 μg/mL) of the total content. The absence of a plateau effect suggests that the degradation process was not completed within the 24 h. This significantly reduced biodegradation rate, compared to unmodified HA, can be considered an advantage for HA–Orn’s future clinical applications and for the development of delivery systems.

### 3.4. MTS Cytotoxicity Assay

**Figure 8:**
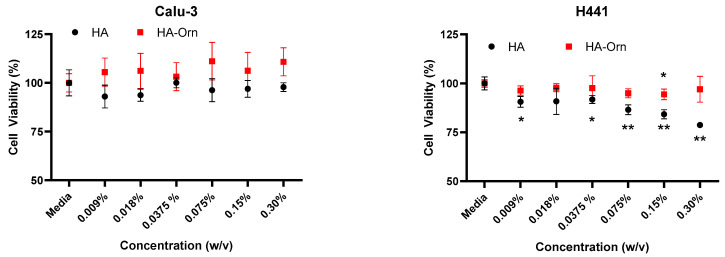
Cell viability was expressed as a percentage of control for Calu-3 and H441 cell lines after 24 h of exposure to HA and HA–Orn using MTS assay (*n* = 3; mean ± St Dev). Statistical differences between the control and treatments were determined using an unpaired t-test with Welch’s correction (GraphPad Prism), and significance was considered when *p* < 0.05 (* *p* < 0.05; ** *p* < 0.01).

The use of HA, as treatment of inflammatory airway pathologies, is well known [7,8,9,10] as previously described. For this reason, an in vitro study was conducted on lung adenocarcinoma-derived epithelial Calu-3 and H441, representative of both the upper and lower lung regions to evaluate the safety of the new product for possible respiratory applications. The cytotoxicity of HA and HA–Orn polymers was evaluated at different concentrations by MTS assay. As shown in Figure 8, no cytotoxic effects were observed on Calu-3 cells exposed up to 0.3% of both formulations, whereas a significant decrease was observed in H441 cells treated with HA from 0.009%. However, exposure of H441 to the HA–Orn polymer showed no cytotoxic effects up to 0.075%. Overall, these results indicate that Calu-3 cells are more tolerant to the HA and HA–Orn polymers than H441 and that H441 cells are more sensitive to HA than to the HA–Orn polymer. Thus, HA–Orn could be considered for targeting the lower regions of the lung.

## 4. Conclusions

In summary, we designed and synthesized a new hyaluronic acid derivative cross-linked by an ornithine derivative. This material was further characterized for its physicochemical characteristics, cytotoxicity, and improved resistance to enzymatic degradation. Using CDMT as an activating agent and the amino acid Ornithine methyl ester as a cross-linking agent, we obtained a new product confirmed by the formation of the new C=O amide bond, with a satisfactory degree of modification. HA–Orn showed a rheological profile attributable to an hydrogel, with a swelling ratio that can be modulated by pH. HA–Orn is an interesting new material that could be utilized either as an active molecule or as an innovative polymeric system for drug delivery. The significant safety profile observed in lung cells was the starting point for the ongoing anti-inflammatory tests of HA–Orn on lung cells to evaluate its potential use as an adjuvant treatment for lung delivery. This behavior is currently under investigation, and ongoing studies will hopefully confirm the above stated potential.

## 5. Patents

This work was filed as patent application N° 102019000024117 on 16 December 2019 by the University of Ferrara.

## Data Availability

The data presented in this study are available within the article and Supplementary Materials.

## Supplementary Materials

The following are available online at https://www.mdpi.com/article/10.3390/pharmaceutics13101672/s1, Figure S1: DVS isotherms of two cycles of moisture sorption and desorption of HA (black lines) and HA–Orn (red lines), Figure S2: Glucuronic acid released from in vitro degradation of HA and HA–Orn in PBS, pH 7.4 at 37 °C in the presence of 50 U/mL of hyaluronidase.
