# Supplementary material for: Design, Synthesis, Characterization, and In Vitro Evaluation of a New Cross-Linked Hyaluronic Acid for Pharmaceutical and Cosmetic Applications
Source: Pharmaceutics. 2021 Oct 13;13(10):1672. doi: 10.3390/pharmaceutics13101672 (PMC8540713; doi:10.3390/pharmaceutics13101672)
Supplement: Supplementary file 1 [file pharmaceutics-13-01672-s001.zip › pharmaceutics-1393809-supplementary.pdf]

pharmaceutics

Supplementary Materials: Design, Synthesis, Characterization, and In Vitro Evaluation of a New Cross-Linked Hyaluronic Acid for Pharmaceutical and Cosmetic Applications

Sabrina Sciabica, Giovanni Tafuro, Alessandra Semenzato, Daniela Traini, Dina Silva, Larissa Gomes Dos Reis, Luisa Canilli, Massimo Terno, Elisa Durini, Silvia Vertuani, Anna Baldisserotto and Stefano Manfredini

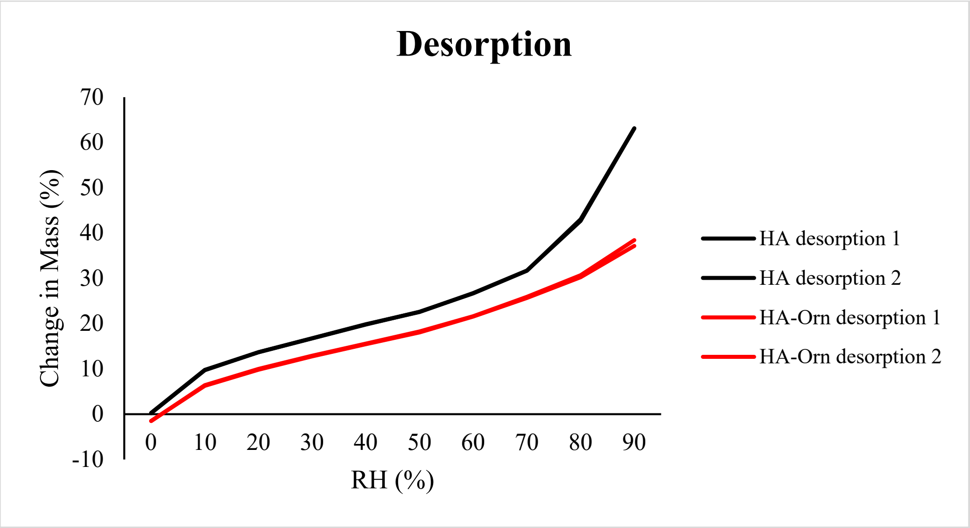

Figure S1. DVS isotherms of two cycles of moisture sorption and desorption of HA (black lines) and HA-Orn (red lines).

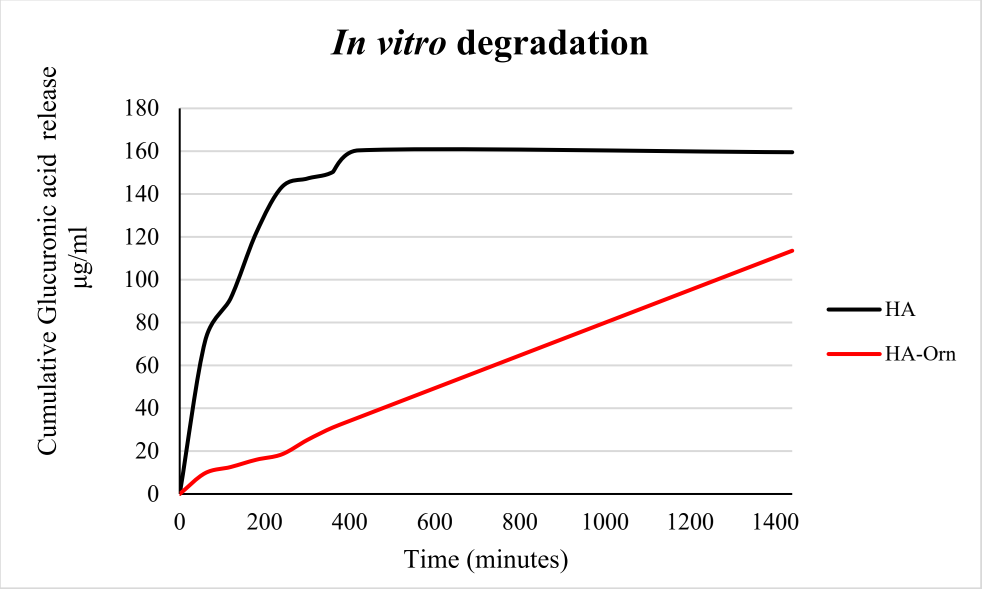

Figure S2. Glucuronic acid released from in vitro degradation of HA, and HA-Orn in PBS, pH 7.4 at 37 °C in the presence of 50U/ml of hyaluronidase.
